# Human activities reshape the spatial overlap between North Chinese leopard and its wild ungulate prey

**DOI:** 10.1186/s12983-024-00545-z

**Published:** 2024-09-26

**Authors:** Yidan Wang, Mingzhang Liu, Fan Xia, Sheng Li

**Affiliations:** 1 State Key Laboratory of Protein and Plant Gene Research, School of Life Sciences, Peking University, Beijing, 100871 China; 2 Institute of Ecology, Peking University, Beijing, 100871 China; 3 National Natural History Museum of China, Beijing, 100050 China

**Keywords:** Large mammals, Interspecific interaction, Camera-trapping, Two-species occupancy model, Niche segregation, Human shelter

## Abstract

**Background:**

Rapidly expanding human activities have profoundly changed the habitat use of both large carnivores and their prey, but whether and how human activities affect the interactions between them has received relatively less attention. In this study, we conducted a systematically designed camera-trapping survey on an endangered large carnivore (North Chinese leopard *Panthera pardus japonensis*) and its wild ungulate prey (Siberian roe deer *Capreolus pygargus* and wild boar *Sus scrofa*) in the Taihang Mountains of central North China. Using conditional two-species occupancy model based on data derived from the extensive sampling effort (15,654 camera-days at 102 camera sites), we examined the relationship of spatial use between leopards and each prey species under the effects of human presence, free-ranging cattle, roads and settlements.

**Results:**

Humans and cattle had contrasting effects on the relationship of spatial use between leopard and roe deer, with higher and lower spatial segregation between them at human and cattle-frequented sites, respectively. Roads might create a shelter for wild boar from leopard predation, with less spatial segregation between them at sites close to the roads.

**Conclusions:**

Our findings demonstrate that human activities are reshaping the spatial overlap between large carnivores and their prey, and have non-equivalent effects among different types of human activity. Such effects may further alter the strength of interspecific interactions between predator and prey, with far-reaching influences on the community and ecosystem that require more research.

**Supplementary Information:**

The online version contains supplementary material available at 10.1186/s12983-024-00545-z.

## Background

As human activities rapidly expand, available habitats free-from disturbance are becoming more limited for wildlife. As a result, an increasing number of wild animals have to share habitat with humans [1, 2], with shifts in habitat use as a common form of behavioral adaption for wildlife inhabiting human-dominated landscapes [3, 4]. Such shifts can facilitate human-wildlife co-existence by allowing wildlife to avoid landscape features where human activities are concentrated (e.g., roads, settlements) or to take advantage of human subsidies (e.g., food waste, agriculture resources). However, when different species differ in their responses to human activities, such shifts may further change their spatial overlap, and also the strength of interspecific interactions [5, 6]. Interactions between predator and prey and the resulting anti-predator behaviors in prey species are crucial in shaping community structures and ecosystem functions [7]. Human effects on the interactions between predators and prey can therefore have far-reaching effects on the community and ecosystem through multiple pathways, such as trophic cascades and suppression of dominant prey [8–10].

By changing the habitat use of both predator and prey, human activities can either increase or decrease the spatial overlap between both parties, thus changing their probability of encounter [11]. Previous studies suggest that when predator and prey are mutually avoiding or attracted by human activities, the probability of predator–prey encounters increases, which potentially skews the behavioral response race in favor of the predator [12, 13]. On the contrary, when predator and prey exhibit opposite responses to human activities, the probability of predator–prey encounters decreases, which potentially skews the behavioral response race in favor of prey [14, 15]. Both human-induced increase and decrease in spatial overlap between predator and prey have been documented in various ecosystems, such as the well-known “human trap” and “human shelter” effects. For example, Fleming and Bateman summarized examples of predators that benefit from anthropogenic environments to increase their hunting success [16]. In North America, the moose (*Alces alces*) were found to use paved roads to shield against traffic-averse brown bears (*Ursus arctos*) [17].

However, different types of human activity can have non-equivalent or even opposite effects on wildlife habitat use [18–20], which are often overlooked in previous studies examining human effects on predator–prey interactions. Humans can kill wildlife more efficiently than predators in natural ecosystems and are considered a “super-predator” [21]. Many animals therefore show a strong fear of human presence, which often leads to behavioral avoidance [22, 23]. Compared to mere presence of humans, sustained, high-intensity disturbance associated with long-term land-use change, such as human infrastructures, may have a more complex effect on wildlife, with ability to simultaneously extirpate wildlife by degrading natural habitats and benefit wildlife by offering human subsidies [19]. Both types of disturbance can occur simultaneously in the landscape, and some animals may avoid direct encounters with humans but at the same time take advantage of subsidies from human infrastructure [23, 24]. Similarly, human effects on predator–prey interactions may also differ with type of human activity. For example, the hypothesis of hunting-mediated predation facilitation suggested that avoidance of human hunters may constrain habitat use of prey, leaving prey with less refuge from natural predators [25, 26], while human infrastructure may create shelters with less predation risk for prey [27]. Disentangling the concurrent effect of multiple types of human activity is therefore crucial for thoroughly understanding human effects on predator–prey interactions and the following ecological consequences.

North China is the political and cultural center of China, with intensive human activities including urbanization, road traffic and agriculture [28]. The endangered North Chinese leopard (*Panthera pardus japonensis*) is the only large carnivore inhabiting the human-disturbed landscapes in Taihang Mountains of central North China [29–31]. Habitat use of both North Chinese leopard and its major wild prey (Siberian roe deer *Capreolus pygargus* and wild boar *Sus scrofa*) are affected by human activities, with effects differing with the type of human activity and species [32, 33]. However, whether and how human activities affect the spatial overlap between North Chinese leopard and its prey is still unknown.

Here, to determine the effects of different types of human activities on the predator–prey interactions, we conducted a camera-trapping survey in Taihang Mountains of central North China. With an extensive dataset collected from 102 camera sites, we built two-species occupancy models to examine (1) whether the occurrence of roe deer and wild boar were dependent on the presence of North Chinese leopard, and (2) whether and how four types of human activity (human presence, free-ranging cattle, road and settlement) affected the relationship of spatial use between North Chinese leopard and its wild prey. We hypothesized that co-occurrence of human and North Chinese leopard at the same site might amplify the spatial avoidance of the two ungulates, as both ungulates showed fearful responses (i.e., increased flight probability and decreased activity) to human sounds in our study area [34]. On the contrary, roads and settlements might create human shelter for the two ungulates as previous studies showed that North Chinese leopard tended to avoid habitats near human infrastructures in our study area, while the two ungulates did not [33].

## Methods

### Study system

**Fig. 1:**
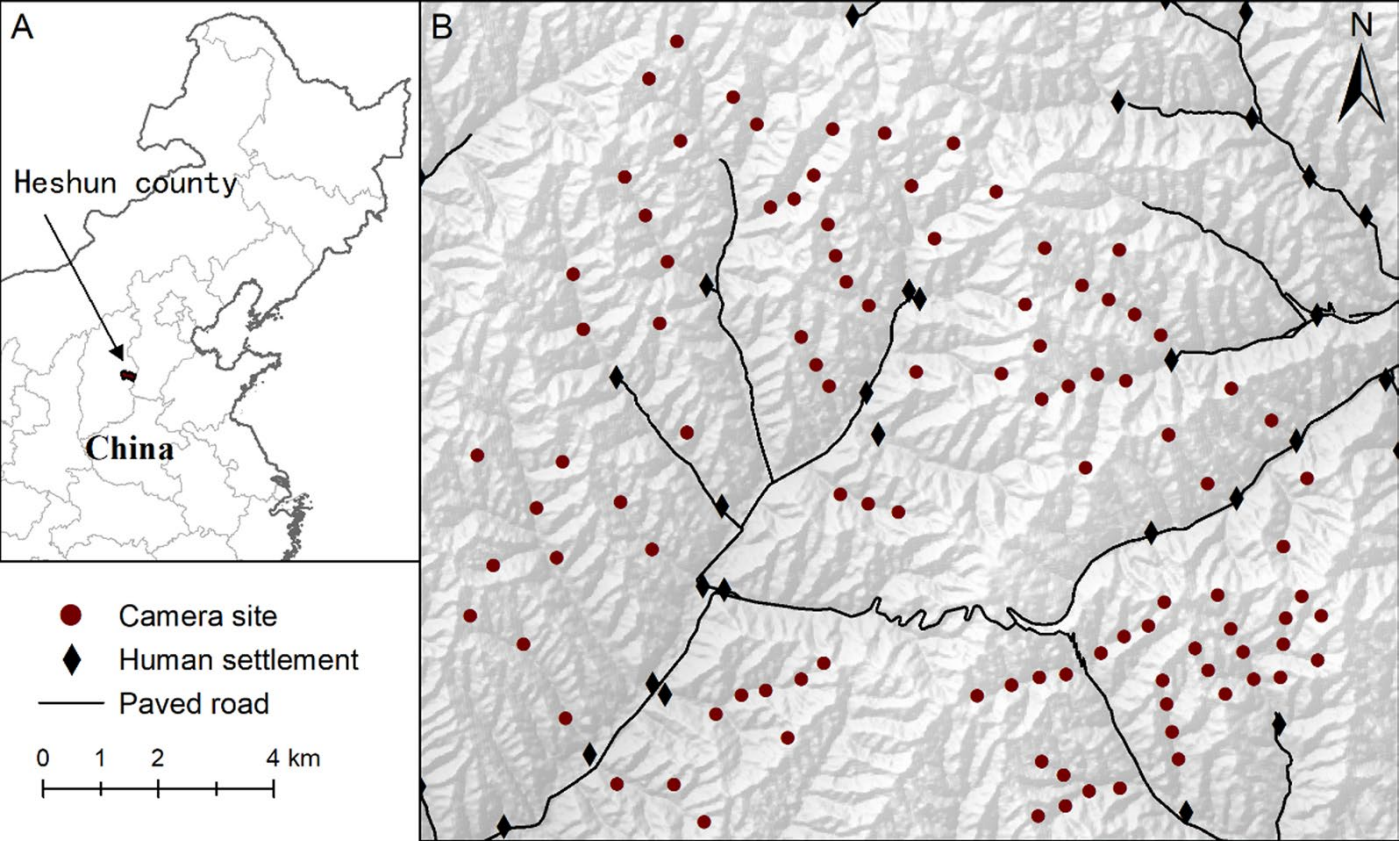
Location of the study area in China (**A**), and the camera sites (n = 102) within the study area (**B**)

We conducted this study in Heshun County of Shanxi Province (113° 15′–113° 25′ E, 37° 15′ –37° 22′ N), which is located in the central Taihang Mountains of North China. The terrain is characterized by rolling hills, with an elevation ranging from 1300 to 1700 m. This area had a temperate continental climate, with an average annual temperature of 7.5 °C and an average annual precipitation of 600 mm. The majority of precipitation happens during the summer months of July and August [35]. The main vegetation type is secondary conifer-deciduous mixed forest, dominated by pine (*Pinus tabuliformis*), oak (*Quercus wutaishanica*), aspen (*Populus davidiana*), and birch (*Betula platyphylla*). North Chinese leopard is the only large carnivore in the study area with Siberian roe deer and wild boar as its main wild prey [36]. The habitat use of leopard, roe deer and wild boar are influenced by multiple types of human activity. Human infrastructures mainly include paved roads and human settlements (Fig. 1), while local residents often use the forests for collecting of non-timber resources (e.g., mushroom, medicine herb and fuel wood) and leave their cattle roaming freely during the day.

### Camera-trapping survey

To determine the effects of human activities on spatial relationships between leopards and their prey, we conducted a systematically designed camera-trapping survey from September 2022 to April 2023 (i.e., the non-growing season) at sites with different intensities of human activities. With no prior knowledge about the presence of humans and cattle, we set cameras along a gradient of distance to the nearest human infrastructure. Specifically, we set all cameras at trails along gullies or ridges starting from roads or settlements. Cameras were separated from each other by at least 500 m (mean = 7.1 km, ranging from 507 to 15.5 km) to ensure the detection probabilities of animals was independent of each other at different camera sites [37]. At each camera site, one motion-triggered camera (CANGLU S1, Qingdao Yequ Nature Technology Co., Ltd., Qingdao, China) was attached to trees at a height of 0.3–0.9 m above the ground along the trail to maximum the detection of the target species. No bait or lures were used during the survey. The cameras were set to active all day, with a 3-min delay between consecutive triggers. The camera took 3 consecutive photos and a 10-s video upon each trigger. We inspected the cameras every 3 months to replace batteries and to retrieve the data. Overall, 102 camera sites were surveyed for an extensive sampling effort of 15,654 camera-days (mean = 153 per site, standard deviation = 18), with distance to the nearest roads or settlements ranging from 305 to 4142 m.

### Data analysis

#### Intensity of human activities

For each camera site, we measured the intensity of four types of human activity, including the relative abundance index (RAI) of human (RH) and cattle (RC), and the distance to the nearest settlement (DS) and road (DR). We considered consecutive images of the same species (i.e., cattle or human) captured within 30 min at the same camera site as one independent detection [38]. The frequency of independent detections, indicating whether a site was less or more likely to be visited by a particular species, can be used as a measure of intensity of use [33, 39]. Therefore, we used the relative abundance index (RAI, average number of independent detections per 100 camera-days) [38] of human and cattle, respectively, at each site to measure their intensity of habitat use. We calculated the Euclidean distance from each camera site to the nearest settlement and paved road, respectively, using ArcMap 10.8. Since the distributions of RH and RC were right-skewed, we log-transformed these two covariates. We also checked all covariates for potential collinearity using the Pearson correlation test. There was no correlation between any covariates (Pearson correlation index < 0.6) [40]. We applied a standardized scale ($\overline{x}$ = 0 and SD = 1) to all covariates to facilitate both model convergence and comparison between their coefficient estimations.

#### Two-species occupancy model

We used a conditional two-species occupancy model to examine (1) whether the detection probability of roe deer and wild boar was conditional on leopard, and (2) whether and how human activities affected the spatial relationship between leopard and the two prey species. We built two-species occupancy models following the framework presented by Richmond et al. (2010) [41]. As originally formulated, the occupancy modelling developed by MacKenzie et al. (2002) [42] depends on an assumption that an individual using a given camera site will always be present and available for sampling. However, such assumption can be violated in camera-trapping surveys as the area that a camera can sample is often smaller than the home ranges of the study species [43, 44]. Therefore, the occupancy ($\psi$) and detection probability ($p$) are often interpreted as proxies for the probability of occurrence (i.e., whether a given species occurred at a site during sampling) and intensity of use (i.e., the overall activity of a species at a given site) respectively [19]. The co-occupancy model developed by Richmond et al. (2010) [41] further assumes that species A is dominant and species B is subordinate, and the occupancy and detection probability of species B could be conditional on the occupancy and detection status of species A (see Table 1 for detailed descriptions of the parameters used in the two-species occupancy model).

**Table 1:** Descriptions of the parameters used in the two-species occupancy model proposed by Richmond et al. (2010) [41]

Parameter	Description
$\psi^{{\text{A}}}$	Occupancy probability for species A
$\psi^{{\text{B}}}$	Occupancy probability for species B
$p^{{\text{A}}}$	Detection probability for species A
$p^{{\text{B}}}$	Detection probability for species B, given species A is absent
$r^{{{\text{BA}}}}$	Detection probability for species B, given both species are present and species A is detected
$r^{{{\text{Ba}}}}$	Detection probability for species B, given both species are present and species A is not detected

Species A is assumed to be dominant, and species B subordinate

In this study, we assumed that the North Chinese leopard is the dominant species (i.e., species A), while ungulate prey (i.e., roe deer and wild boar) are subordinate (i.e., species B). We noticed that the naïve occupancy, defined as the proportion of camera sites where a species was detected at least once during the survey without accounting for the imperfect detection [45], was 0.92 for roe deer and 0.93 for wild boar in this study area (Table 2). When accounting for the imperfect detection, this suggested that the probability of occurrence must be 1 for at least 92% and 93% of the camera sites for roe deer and wild boar, respectively, and were unlikely to be affected by leopards. Therefore, for each species pair (i.e., leopard-roe deer, leopard-wild boar), we compared the performance of the conditional two-species occupancy model under the following four hypotheses to examine whether the intensity of use of roe deer and wild boar was affected by leopards:

1. Detection probability of species B was independent of species A (i.e., $p^{{\text{B}}} = r^{{{\text{Ba}}}} = r^{{{\text{BA}}}}$);
2. Detection probability of species B was conditional on the presence of species A (i.e., $p^{{\text{B}}} \ne r^{{{\text{Ba}}}} = r^{{{\text{BA}}}}$);
3. Detection probability of species B was conditional on the detection of species A (i.e., $p^{{\text{B}}} = r^{{{\text{Ba}}}} \ne r^{{{\text{BA}}}}$);
4. Detection probability of species B was conditional on both the presence and detection of species A (i.e., $p^{{\text{B}}} \ne r^{{{\text{Ba}}}} \ne r^{{{\text{BA}}}}$).

We defined 10 consecutive camera days at each camera site as a survey occasion following previous study on the North Chinese leopard and sympatric mammals in North China [32, 46]. We recorded whether a given species was detected (1) or not (0) in each occasion. The detection of the North Chinese leopard during a certain occasion at a given site was drawn from the following Bernoulli distribution:

$$D^{{\text{A}}} { }\sim { }bern\left( {p^{{\text{A}}} \times z^{{\text{A}}} } \right),$$

Here, $z^{{\text{A}}}$ is the actual occupancy state (1 or 0) of the North Chinese leopard at the given camera site, which is drawn from the following Bernoulli distribution:

$$z^{{\text{A}}} { }\sim { }bern\left( {\psi^{{\text{A}}} } \right)$$

If the detection probability of species B was assumed to be independent of species A, its detection ($D^{{\text{B}}}$) was modeled as

$$D^{{\text{B}}} { }\sim { }bern\left( {p^{{\text{B}}} \times z^{{\text{B}}} } \right)$$

If the detection probability of species B was conditional on the presence of species A, its detection was modeled as

$$D^{{\text{B}}} { }\sim { }bern\left( {p^{{\text{B}}} \times z^{{\text{B}}} \times \left( {1 - z^{{\text{A}}} } \right)} \right) + bern\left( {r^{{{\text{Ba}}}} \times z^{{\text{B}}} \times z^{{\text{A}}} } \right)$$

If the detection probability of species B was conditional on the detection of species A, its detection was modeled as

$$D^{{\text{B}}} { }\sim { }bern\left( {r^{{{\text{Ba}}}} \times z^{{\text{B}}} \times \left( {1 - D^{{\text{A}}} } \right)} \right) + bern\left( {r^{{{\text{BA}}}} \times z^{{\text{B}}} \times D^{{\text{A}}} } \right)$$

If the detection probability of species B was conditional on both the presence and detection of species A, its detection was modeled as

$$D^{{\text{B}}} { }\sim { }bern\left( {p^{{\text{B}}} \times z^{{\text{B}}} \times \left( {1 - z^{{\text{A}}} } \right)} \right) + bern\left( {r^{{{\text{Ba}}}} \times z^{{\text{B}}} \times z^{{\text{A}}} \times \left( {1 - D^{{\text{A}}} } \right)} \right) + bern\left( {r^{{{\text{BA}}}} \times z^{{\text{B}}} \times z^{{\text{A}}} \times D^{{\text{A}}} } \right),$$

where $z^{{\text{B}}}$ is the actual occupancy state (1 or 0) of the ungulates at the given camera site, which is drawn from the following Bernoulli distribution:

$$z^{{\text{B}}} { }\sim { }bern\left( {\psi^{{\text{B}}} } \right)$$

We formulated the occupancy and detection probability using logistic regression models. Previous study suggested that human activities and other environmental variables had limited effect on the occupancy of the study species, especially for the two prey with naïve occupancy close to 1 [47, 48]. Therefore, we included the latitude and longitude of each camera site as the occupancy variables to address the possible spatial autocorrelation in occupancy probabilities. For the detection probability, we used DR, DS, RH and RC as detection variables to examine how human activities affected the intensity of habitat use of each species.

We analyzed the two-species occupancy models in a Bayesian framework using the JAGS language called through the package *R2jags* in R (v.4.2.2; R Core Team, 2022) [49]. For each model, we ran three Markov Chain Monte Carlo (MCMC) chains of 100,000 iterations each and made inference from 25,000 samples from the posterior distribution of each chain after burn in of 50,000 and a thinning rate of 5. We chose vague priors for all variables. We confirmed the convergence of MCMC chains by visually inspecting trace plots via the Gelman-Rubin statistic ($\hat{R}$) (all $\hat{R}$ < 1.1, see Additional file 1: Table S1 and S2) [50].

We assessed the performance of the models based on different hypotheses by calculating the expected log pointwise predictive density (elpd) of each model through the package *loo* in R (v.4.2.2; R Core Team, 2022) [51]. A higher elpd value indicates better model performance, and a difference in elpd ($\Delta {\text{elpd}}$) of more than 4 between two models suggests a notable difference in their performance [52]. For models with similar performance, we considered the simpler one as the better one based on the principle of parsimony. We further used Moran’s *I* based on the site-sum residuals of the detection probability through the package *spdep* in R (v.4.2.2; R Core Team, 2022) to assure that there was no significant spatial autocorrelation in the models with best performances [53, 54].

We considered variables significant and marginally significant when the 95% and 85% Bayesian credible intervals (BCIs) of their coefficient estimates did not overlap with zero, respectively. When model comparison suggested that detection probabilities of roe deer and wild boar were conditional on the presence of leopard, we calculated how their relationship to each detection variable (i.e., the coefficient estimates) differed in the presence and absence of leopards. A statistically significant difference (i.e., 95% or 85% BCIs do not overlap with zero) indicates that prey respond differently to human activities in the presence and absence of the leopard. Besides, we predicted the detection probabilities for the roe deer and wild boar, respectively, using the mean values of each variable (DR, DS, RH and RC) across all camera sites to compare the differences in detection probabilities of the two species in the presence or absence of leopards in the study area.

Finally, we calculated the species interaction factor (SIF) proposed by Richmond et al. (2010) [41] to measure the relationships of spatial use between leopard and each of the two ungulates using the following formula:

$${\text{SIF}} = { }\frac{{r^{B} }}{{r^{B} \times \psi^{{\text{A}}} + p^{B} \times \left( {1 - \psi^{{\text{A}}} } \right)}}$$

If the habitat use of prey was independent of the leopard, then the SIF is equal to one, while an SIF less or greater than one indicates that species B is less or more likely to be detected with species A than expected under a hypothesis of independence, respectively.

## Results

**Fig. 2:**
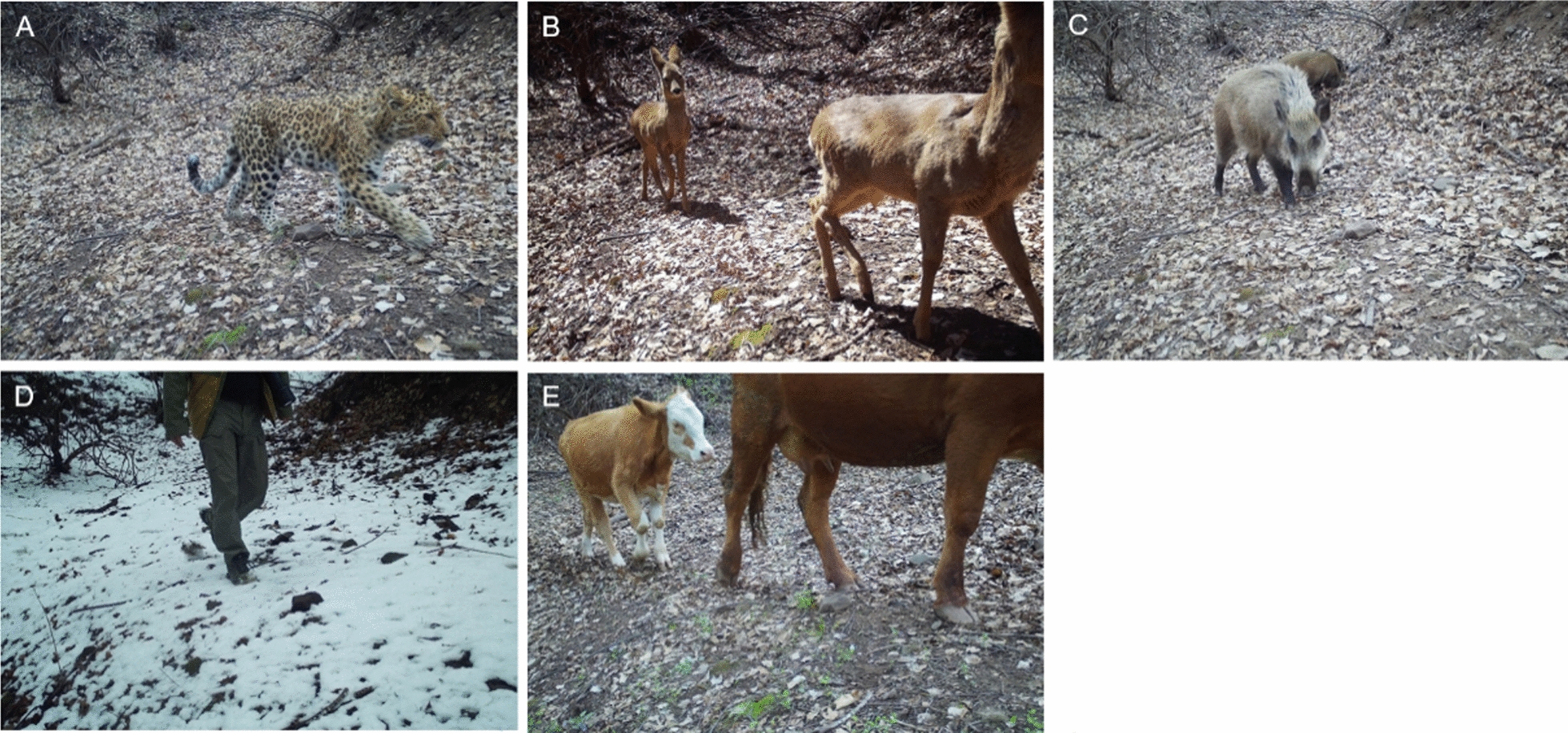
Photographs of leopard (**A**), Siberian roe deer (**B**), wild boar (**C**), human (**D**) and domestic cattle (**E**) captured at the same camera site during the camera-trapping survey from September 2022 to April 2023 in Taihang Mountains, central North China

In total, we obtained data from 102 cameras with a total effort of 15,654 camera-days (Table 2). Leopards were detected at 40 camera sites with 106 independent detections and a RAI of 0.68. Roe deer, wild boar, human and cattle were detected at 92%, 93%, 100% and 55% of all camera sites with a RAI of 8.19, 7.77, 1.51, and 5.24, respectively (Fig. 2).

**Table 2:** Number of independent detections, relative abundance index and naïve occupancy of leopard, Siberian roe deer, wild boar, human and domestic cattle during the camera-trapping survey from September 2022 to April 2023 in Taihang Mountains, central North China

Species	Scientific name	No. of independent detections	RAI	Naïve occupancy
Leopard	*Panthera pardus japonensis*	106	0.68	0.39
Siberian Roe deer	*Capreolus pygargus*	1282	8.19	0.92
Wild boar	*Sus scrofa*	1217	7.77	0.93
Human	*Homo sapiens*	236	1.51	1
Cattle	*Bos taurus*	821	5.24	0.55

**Fig. 3:**
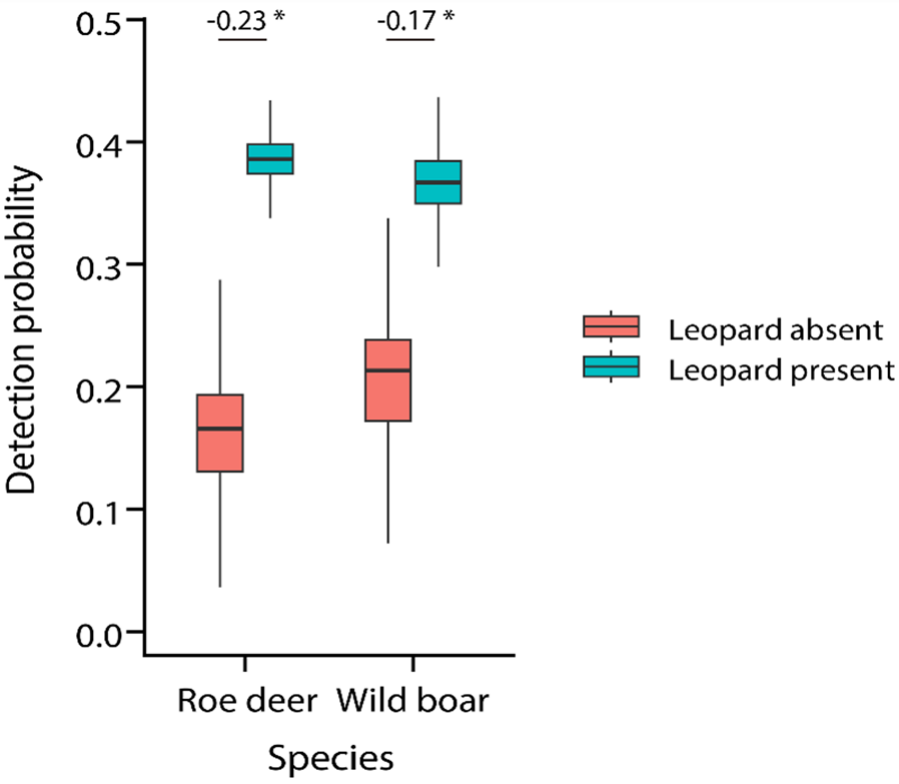
Model-estimated detection probabilities of Siberian roe deer and wild boar with the presence and absence of leopard using mean values for DR and DS, RH and RC across all camera sites. Vertical lines extending above and below the boxes represent 95% Bayesian credible intervals. The numbers above the boxes are differences between the predicted detection probability (leopard absent minus leopard present), with a star symbol indicating statistical significance (i.e., 95% Bayesian credible interval does not overlap with zero)

Model comparison suggested that the detection probabilities of both roe deer and wild boar were conditional on the presence of leopard (Table 3), with detection probabilities of roe deer and wild boar significantly higher at leopard-present sites than leopard-absent sites (roe deer: difference = − 1.28, 95% BCI [− 2.38, − 0.556]; wild boar: difference = − 0.895, 95% BCI [− 1.89, − 0.234]) (Additional file 1: Table S1, S2, Fig. 3, Additional file 1: Fig. S1). The Moran’s *I* values for the site-sum residuals of the detection probabilities from the models with best performances for both roe deer (*I* = − 0.002, p-value = 0.289) and wild boar (*I* = − 0.023, p-value = 0.821) indicated that there was no statistically significant spatial autocorrelation.

**Table 3:** Rankings of conditional two-species models of Siberian roe deer and wild boar based on four hypotheses

Model	Hypothesis	$\Delta {\text{elpd}}$	$\Delta {\text{se}}$
*Siberian roe deer*			
$p^{{\text{B}}} \ne r^{{{\text{Ba}}}} = r^{{{\text{BA}}}}$	2	0.00	0.00
$p^{{\text{B}}} \ne r^{{{\text{Ba}}}} \ne r^{{{\text{BA}}}}$	4	− 4.18	2.68
$p^{{\text{B}}} = r^{{{\text{Ba}}}} = r^{{{\text{BA}}}}$	1	− 23.87	15.31
$p^{{\text{B}}} = r^{{{\text{Ba}}}} \ne r^{{{\text{BA}}}}$	3	− 28.95	15.31
*Wild boar*			
$p^{{\text{B}}} \ne r^{{{\text{Ba}}}} = r^{{{\text{BA}}}}$	2	0.00	0.00
$p^{{\text{B}}} \ne r^{{{\text{Ba}}}} \ne r^{{{\text{BA}}}}$	4	− 3.44	2.92
$p^{{\text{B}}} = r^{{{\text{Ba}}}} = r^{{{\text{BA}}}}$	1	− 17.54	11.63
$p^{{\text{B}}} = r^{{{\text{Ba}}}} \ne r^{{{\text{BA}}}}$	3	− 21.36	11.23

Inference about best fitting models were based on the difference of the expected log pointwise predictive density (∆elpd)

**Fig. 4:**
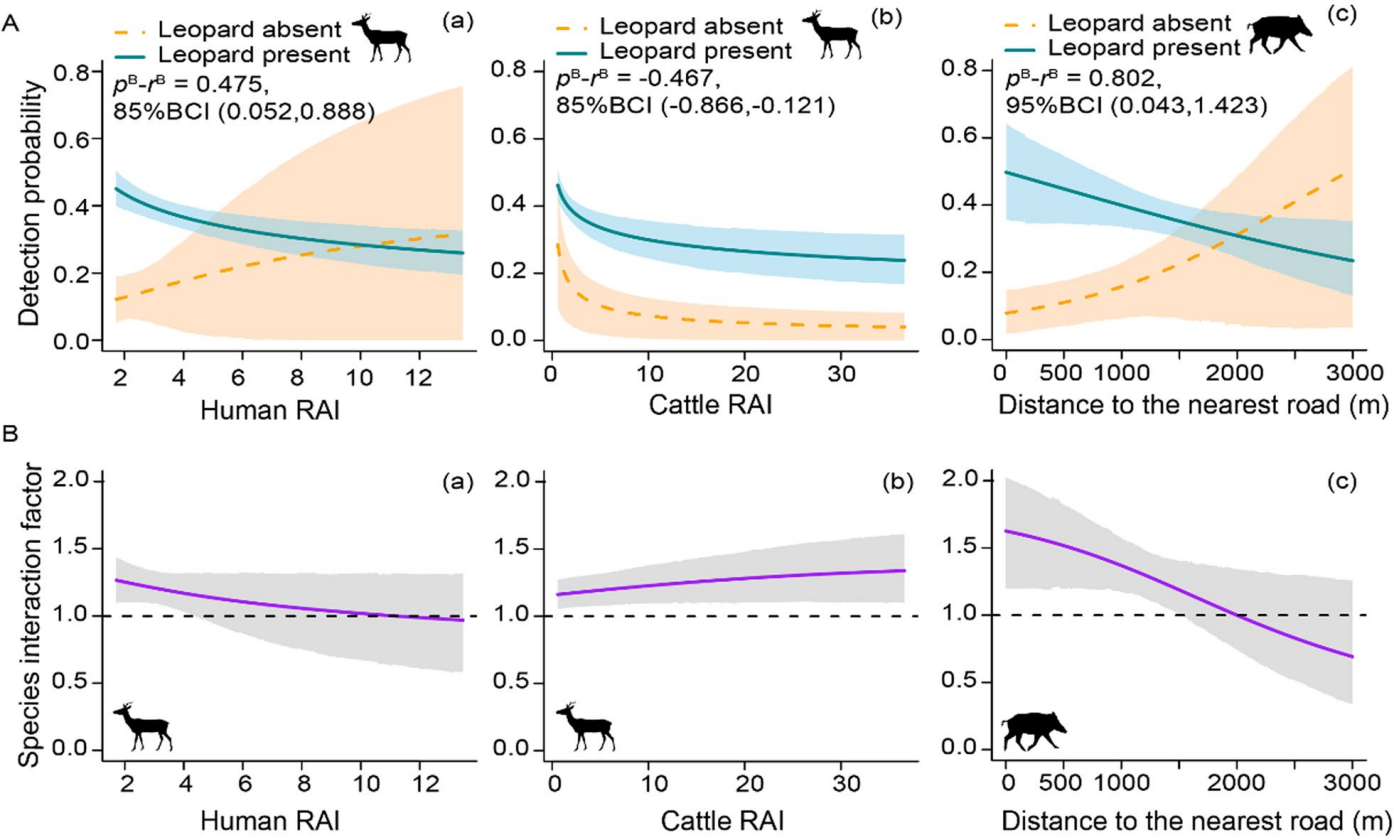
The detection probability of two ungulate prey (**A**) and the species interaction factors between leopard and its prey (**B**) affected by three types of human activities (a-human, b-cattle, c-road). Lines and shaded areas indicate mean and 95% Bayesian credible intervals (BCIs) of the predictions. Here we only show human activities with significant and marginally significant differences (*p*^*B*^—*r*^*B*^) in their relationship with detection probabilities of the prey when leopards are absent and present (i.e., the 95% and 85% BCIs of the estimated differences have no overlap with zero)

Model estimates showed that the detection probability of roe deer had marginally significant differences in its relationship with RH and RC when leopards were present or absent (RH: difference = 0.475, 95% BCI [− 0.5, 1.44]; RC: difference = − 0.467, 95% BCI [− 1.11, 0.064]) (Additional file 1: Table S1, Fig. S1-A). Specifically, the detection probability of roe deer tended to increase with RH when leopards were absent ($p^{{\text{B}}}$(RH) = 0.246, 95% BCI [− 0.727, 1.161]), but significantly decreased with RH when leopards were present ($r^{{\text{B}}}$(RH) = − 0.229, 95% BCI [− 0.357, − 0.11]) (Additional file 1: Table S1, Fig. 4A-a), with SIF between leopards and roe deer decreasing when RH increased (Fig. 4B-a). The detection probability of roe deer had a less negative relationship with RC when leopards were present ($p^{{\text{B}}}$(RC) = − 0.79, 95% BCI [− 1.497, − 0.244]; $r^{{\text{B}}}$(RC) = − 0.323, 95% BCI [− 0.482, − 0.163) (Additional file 1: Table S1, Fig. 4A-b). Contrary to its relationship with RH, the SIF between leopard and roe deer increased when RC increased (Fig. 4B-b).

For wild boars, detection probability had a significantly different relationship with DR when leopards were present or absent (difference = 0.802, 95% BCI [0.043, 1.4]) (Additional file 1: Table S2, Fig. S1-B). Specifically, the detection probability of wild boar was higher near roads when leopards were present ($r^{{\text{B}}}$(DR) = − 0.258, 95% BCI [− 0.492, − 0.002]) but lower near roads when leopards were absent ($p^{{\text{B}}}$(DR) = 0.544, 95% BCI [− 0.114, 1.083]) (Fig. 4A-c), with SIF between leopard and wild boar decreased when DR increased (Fig. 4B-c).

## Discussion

Although a growing body of research has widely demonstrated the human effects on wildlife habitat use [19, 55, 56], whether and how human activities will affect spatial overlap and strength of interspecific interactions between predator and prey have received less attention. Based on the conditional two-species occupancy model, our analyses suggest that human activities had different effects on the habitat use of prey species when leopards were present or absent, thus altering the relationship of spatial use between the predator and prey.

Overall, the model-estimated detection probabilities of prey with average level of human activities in our study area suggest that, in the non-growing season, both roe deer and wild boar were more likely to be detected with leopards than expected under a hypothesis of independence (Fig. 3). More important, our analyses further indicate that these patterns reflect the interactions under the effects of human activities, which may be different from those without human influence. Specifically, for wild boar, the SIF decreased as distance to roads increased, with SIF smaller than 1 when DR was greater than 2000 m. This result suggests that wild boars are less likely to be detected with leopards at sites far from roads. The overall spatial relationship between leopard and wild boar, however, is the result of an average distance to roads of 1300 m across all camera sites in our study area. The opposite patterns of spatial relationship between leopards and wild boar at sites close to or far from roads suggest that roads completely change the spatial overlap between these two species, and probably also their strength of interactions in our study area. For roe deer, their spatial relationship with leopards in our study area was similar to that with low intensity of human activities, suggesting limited human effects on the spatial overlap between these two species. However, as SIF between leopards and roe deer decreased with RH, we speculate that if the intensity of human presence further increased in our study area (for example, in the growing season with more intensive human use of the forests [33]), the interaction between leopards and roe deer may also be reshaped.

Our analyses showed that wild boar avoided roads when leopards were absent, but preferred roads when leopards were present. Such opposite relationship in the absence or presence of leopards suggest that wild boar may explore areas close to roads as a shelter from leopard predation, especially given the fact that leopards tend to avoid roads in their habitat use ($p^{{\text{A}}}$(DR) = 0.15, 95% BCI [− 0.23, 0.49]). On the contrary, roe deer differed in their responses to human and cattle presence. Roe deer increased their avoidance of humans when leopards were present, suggesting that the co-occurrence of humans and leopards may amplify the perceived risks for roe deer. Besides, as the detection probability of leopards was positively correlated to humans ($p^{{\text{A}}}$(RH) = 0.34, 95% BCI [0.151, 0.521]) (Additional file 1: Table S1), it is likely that roe deer can spatially avoid humans and leopards simultaneously, as demonstrated by the “predator attraction” effect suggested by Van Scoyoc et al. (2023) [11]. On the contrary, roe deer decreased their avoidance of cattle when leopards were present. Although cattle can compete with roe deer for space and food resources and increase the risk of parasitic transmission, they can also serve as alternative prey, especially the calves, for leopards [57] and such human-associated prey resources may reduce carnivore predation on the natural prey [58].

Overall, our findings provide an empirical case for the human effects on the relationships of spatial uses between leopards and their wild prey. Previous theoretical frameworks often considered human effects on interspecific interactions as results of separate effects on predator and prey [59], but often overlooked the possibility that prey may respond to human activities differently in the presence or absence of predator. By comparing the spatial responses of prey to human activities when leopards are absent or present, our findings highlight that prey response to human activities may be subject to the habitat use of leopards. Although the effects of single types of human activity (e.g., human presence, or human infrastructure) on predator–prey interactions have been examined separately in previous studies [25–27], comparison between them in the same system has been limited. Our findings of non-equivalent and even opposite effects of human, cattle and road on the spatial relationships between leopards and their prey suggest that different types of human activities differ in their effects on inter-specific interactions. However, the mechanisms mediating such different effects need further exploration. For instance, compared to human presence, cattle can be alternative food resources for leopards, thus reducing leopard predation on roe deer and increasing their spatial overlap [33]. Identifying the dominant human activity in reshaping predator–prey interactions and its functioning mechanism is therefore important for developing adequate management policies to maintain the inter-specific interaction network.

We emphasize that, although human activities can change the spatial relationships between leopards and their prey, the encounter rate and strength of their interactions may also depend on their behaviors on other ecological dimensions. Prey may use sites occupied by predators, but still avoid them temporally or by reactive behavioral responses [34, 60]. Thereby, to thoroughly understand the mechanism determining human effects on the wildlife communities, it is crucial to further examine human effects on the behavior pattern overlap between predator and prey on different ecological dimensions (e.g., time and space) and the predation rate of predators on their prey.

## Conclusion

With the continuous expansion of human activities and increasing human disturbances [61, 62], understanding the potential mechanisms underlying the effects of human activities on wildlife interspecific relationships is pivotal to the development of effective management and conservation policies. Our results confirm that human activity can alter the relationships of spatial uses between predator and prey. Our findings also provide a supportive case for the human shelter effect of roads. Roads are extending into a great proportion of world’s natural habitats, with effects on wildlife behaviors and populations [63, 64]. In addition to effects on single species, we further highlight the need for examining road effects on inter-specific interactions in wildlife communities. Our research demonstrates that direct human encounters and human infrastructures have distinct effects on prey species. We recommend that future studies in assessing human impacts shall consider using multiple metrics rather than a single measurement (e.g., conventionally solely the human presence or human infrastructure) as a proxy for human disturbance. We also highlight that spatial overlap is only a primary proxy for predator–prey interactions. Therefore, further research focusing on wildlife persisting in shared habitats with humans shall integrate results on behavioral responses, physiological status and population dynamics of predators and prey to gain a deeper and more comprehensive insight into human effects on their interactions.

## Supplementary Information

Additional file 1.

## Data Availability

The datasets used in this study are available from the corresponding author on reasonable request.
