# Supplementary material for: Human activities reshape the spatial overlap between North Chinese leopard and its wild ungulate prey
Source: Front Zool. 2024 Sep 26;21:24. doi: 10.1186/s12983-024-00545-z (PMC11426093; doi:10.1186/s12983-024-00545-z)
Supplement: Supplementary file 1 — Additional file 1. [file 12983_2024_545_MOESM1_ESM.docx]

**Supplementary**

**Table S1.** Coefficient estimates for each parameter of the optimal model of the conditional two-species occupancy model for leopard-roe deer.

|  | **mean** | **sd** | **median** | **l95** | **u95** | **Rhat** | **MCEpc** |
| --- | --- | --- | --- | --- | --- | --- | --- |
| $\psi^{A}$(Int) | 0.961 | 0.421 | 0.934 | 0.173 | 1.821 | 1.001 | 1.725 |
| $\psi^{A}$(X) | 0.264 | 0.328 | 0.252 | -0.355 | 0.935 | 1.002 | 1.231 |
| $\psi^{A}$(Y) | -0.232 | 0.306 | -0.233 | -0.835 | 0.363 | 1.002 | 0.879 |
| $\psi^{B}$(Int) | 3.316 | 0.655 | 3.245 | 2.141 | 4.628 | 1.000 | 1.313 |
| $\psi^{B}$(X) | -0.322 | 0.616 | -0.292 | -1.553 | 0.871 | 1.000 | 0.944 |
| $\psi^{B}$(Y) | -0.780 | 0.583 | -0.757 | -1.951 | 0.355 | 0.998 | 1.05 |
| $p^{A}$(Int) | -2.982 | 0.159 | -2.983 | -3.295 | -2.674 | 1.000 | 1.227 |
| $p^{A}$(DR) | 0.150 | 0.181 | 0.158 | -0.230 | 0.490 | 1.001 | 1.063 |
| $p^{A}$(DS) | 0.084 | 0.240 | 0.068 | -0.381 | 0.584 | 1.002 | 1.233 |
| $p^{A}$(RH) | 0.340 | 0.094 | 0.341 | 0.151 | 0.521 | 1.000 | 0.715 |
| $p^{A}$(RC) | -0.170 | 0.154 | -0.168 | -0.471 | 0.133 | 1.000 | 0.751 |
| $p^{B}$(Int) | -1.741 | 0.491 | -1.615 | -2.905 | -1.043 | 1.003 | 2.551 |
| $p^{B}$(DR) | 0.203 | 0.377 | 0.239 | -0.451 | 0.823 | 1.001 | 2.172 |
| $p^{B}$(DS) | -0.328 | 0.338 | -0.330 | -0.967 | 0.327 | 1.001 | 2.285 |
| $p^{B}$(RH) | 0.246 | 0.455 | 0.265 | -0.727 | 1.161 | 1.002 | 2.222 |
| $p^{B}$(RC) | -0.790 | 0.309 | -0.762 | -1.497 | -0.244 | 0.999 | 1.579 |
| $r^{B}$(Int) | -0.465 | 0.075 | -0.465 | -0.615 | -0.321 | 1.002 | 1.34 |
| $r^{B}$(DR) | 0.183 | 0.082 | 0.182 | 0.021 | 0.342 | 0.999 | 0.816 |
| $r^{B}$(DS) | -0.233 | 0.108 | -0.231 | -0.442 | -0.012 | 1.001 | 1.262 |
| $r^{B}$(RH) | -0.229 | 0.063 | -0.228 | -0.357 | -0.110 | 1.001 | 0.776 |
| $r^{B}$(RC) | -0.323 | 0.082 | -0.323 | -0.482 | -0.163 | 1.001 | 1.004 |

Note: $\psi^{A}$ and $\psi^{B}$are the occupancy probability for leopard and roe deer respectively. $p^{A}$ is the detection probability for leopard. $p^{B}$ is the detection probability for roe deer given leopard is absent. $r^{B}$ is the probability for roe deer given leopard is present. The letters inside the parentheses represent the names of the covariates, including intercept (Int), longitude of each camera site (X), latitude of each camera site (Y), distance to the nearest road (DR), distance to the nearest settlement (DS), the relative abundance index of human (RH), and the relative abundance index of cattle (RC). Mean, sd, median, l95 and u95 are the mean value, standard deviance, median value, highest and lowest 95% confidence intervals of the estimation, respectively. Rhat is the Gelman-Rubin statistic, used for confirming the convergence of the model. MCEpc is the percentage of the standard deviance of the posterior distribution.

**Table S2.** Coefficient estimates for each parameter of the optimal model of the conditional two-species occupancy model for leopard-wild boar.

|  | **mean** | **sd** | **median** | **l95** | **u95** | **Rhat** | **MCEpc** |
| --- | --- | --- | --- | --- | --- | --- | --- |
| $\psi^{A}$(Int) | 0.226 | 0.43 | 0.156 | -0.504 | 1.17 | 1.002 | 2.574 |
| $\psi^{A}$(X) | -0.058 | 0.346 | -0.094 | -0.71 | 0.673 | 1.002 | 2.692 |
| $\psi^{A}$(Y) | -0.043 | 0.294 | -0.043 | -0.617 | 0.545 | 1.001 | 1.444 |
| $\psi^{B}$(Int) | 2.643 | 0.685 | 2.524 | 1.521 | 4.032 | 1.001 | 1.868 |
| $\psi^{B}$(X) | 0.829 | 0.502 | 0.789 | -0.107 | 1.869 | 0.999 | 1.558 |
| $\psi^{B}$(Y) | 0.257 | 0.488 | 0.23 | -0.717 | 1.216 | 0.999 | 0.87 |
| $p^{A}$(Int) | -2.586 | 0.228 | -2.567 | -3.05 | -2.166 | 1.001 | 2.244 |
| $p^{A}$(DR) | 0.106 | 0.208 | 0.107 | -0.311 | 0.504 | 1.001 | 1.875 |
| $p^{A}$(DS) | -0.212 | 0.31 | -0.211 | -0.837 | 0.387 | 1.001 | 1.113 |
| $p^{A}$(RH) | 0.252 | 0.108 | 0.253 | 0.042 | 0.469 | 1.002 | 1.07 |
| $p^{A}$(RC) | -0.362 | 0.184 | -0.36 | -0.737 | -0.011 | 1.001 | 0.801 |
| $p^{B}$(Int) | -1.441 | 0.46 | -1.306 | -2.462 | -0.796 | 1.000 | 3.748 |
| $p^{B}$(DR) | 0.544 | 0.302 | 0.565 | -0.114 | 1.083 | 1.006 | 3.167 |
| $p^{B}$(DS) | 0.337 | 0.246 | 0.356 | -0.263 | 0.793 | 1.003 | 2.271 |
| $p^{B}$(RH) | 0.085 | 0.249 | 0.072 | -0.364 | 0.604 | 1.001 | 1.047 |
| $p^{B}$(RC) | -0.202 | 0.269 | -0.199 | -0.743 | 0.287 | 1.002 | 1.685 |
| $r^{B}$(Int) | -0.547 | 0.111 | -0.546 | -0.773 | -0.336 | 1.000 | 1.219 |
| $r^{B}$(DR) | -0.258 | 0.125 | -0.262 | -0.492 | -0.002 | 1.000 | 1.599 |
| $r^{B}$(DS) | 0.056 | 0.243 | 0.072 | -0.421 | 0.515 | 1.003 | 2.866 |
| $r^{B}$(RH) | -0.074 | 0.074 | -0.073 | -0.221 | 0.067 | 1.002 | 1.293 |
| $r^{B}$(RC) | -0.283 | 0.132 | -0.287 | -0.534 | -0.017 | 1.002 | 2.409 |

Note: $\psi^{A}$ and $\psi^{B}$are the occupancy probability for leopard and roe deer respectively. $p^{A}$ is the detection probability for leopard. $p^{B}$ is the detection probability for roe deer given leopard is absent. $r^{B}$ is the probability for roe deer given leopard is present. The letters inside the parentheses represent the names of the covariates, including intercept (Int), longitude of each camera site (X), latitude of each camera site (Y), distance to the nearest road (DR), distance to the nearest settlement (DS), the relative abundance index of human (RH), and the relative abundance index of cattle (RC). Mean, sd, median, l95 and u95 are the mean value, standard deviance, median value, highest and lowest 95% confidence intervals of the estimation, respectively. Rhat is the Gelman-Rubin statistic, used for confirming the convergence of the model. MCEpc is the percentage of the standard deviance of the posterior distribution.

**
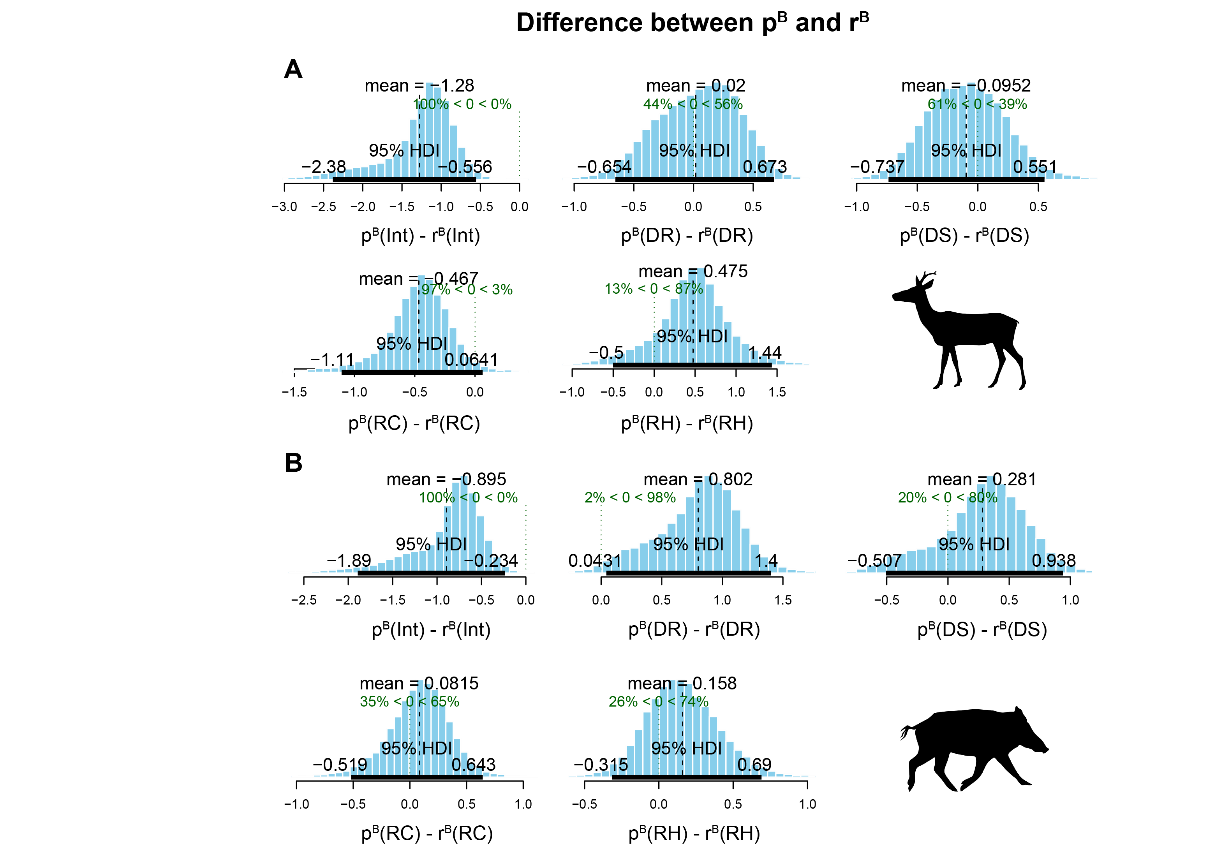
**

**Fig. S1** Difference between the coefficient estimates of each parameter when leopard absent ($p^{B}$) and present ($r^{B}$) for the leopard-roe deer (A) and leopard-wild boar (B) models. Int is the intercept. DR is the distance to the nearest road. DS is the distance to the nearest settlement. RH is the relative abundance index of human. RC is the relative abundance index of cattle. For each subplot, the mean value and the 95% highest density interval (95% HDI) are labeled with black numbers. The green dotted line indicates the position of the number zero, and the corresponding percentages above represent the proportions greater than and less than zero.
